# Assessment of the management of acute myocardial infarction patients and their outcomes at the Nairobi Hospital from January 2007 to June 2009

**DOI:** 10.5830/CVJA-2015-091

**Published:** 2016

**Authors:** Redemptar Kimeu, Charles Kariuki

**Affiliations:** Nairobi Cardiovascular Clinic, Nairobi, Kenya; Nairobi Cardiovascular Clinic, Nairobi, Kenya

**Keywords:** acute myocardial infarction, CAD risk factors, outcomes of acute myocardial infarction, Nairobi Hospital, Kenya

## Abstract

**Introduction:**

The demographics, clinical characteristics and management of patients presenting at the Nairobi Hospital with acute myocardial infarction have not been documented in the past. There is a paucity of studies on this subject in this region.

**Methods:**

A retrospective, hospital-based study was carried out, examining data of patients presenting at Nairobi Hospital with acute myocardial infarction between January 2007 and June 2009. The data collected were patient demographics, coronary artery disease (CAD) risk factors, clinical presentation, GRACE score risk stratification, coronary anatomical findings on angiography, interventions and outcomes during hospitalisation.

**Results:**

Sixty-four patients were recruited (mean age 56.7 years). The CAD risk-factor profile included systemic hypertension in 71.9% of patients, age over 55 or 65 years in men and women, respectively in 42.2%, 35.9% of subjects were smokers, low high-density lipoprotein cholesterol levels in 25%, diabetes mellitus in 25%, family history of premature coronary artery disease in 8%, prior acute coronary syndrome in 18.8%, ST-segment elevation myocardial infarction (STEMI) in 60.9% and non-ST-segment elevation myocardial infarction (NSTEMI) in 39.1% of patients. In the STEMI arm, 79.5% of patients underwent thrombolysis, 17.9% had rescue percutaneous coronary intervention (PCI) and 2.6% had no reperfusion therapy. Medical management was carried out in 29% of the patients, 19.1% had a coronary artery bypass graft and 40.4% had PCI. The mean duration of hospitalisation was 6.69 days. The in-hospital mortality rate was 9.4% and mean in-hospital probability of death according to the GRACE risk score was 16.05%. Discharge medication was a β-blocker in 84.5% of patients, an ACE inhibitor or angiotensin receptor blocker in 48.3%, low-dose aspirin in 96.6%, clopidogrel in 96.6% and statins in 93.1%.

**Conclusion:**

The risk-factor assessment in our population, albeit small, was in keeping with the traditional risk factors for coronary artery disease. There is, however, room for improvement in reconciling the gap between actual and recommended patient care.

## Introduction

The demographic and clinical characteristics of patients presenting with acute myocardial infarction at the Nairobi Hospital have not been documented in the past. Abnormal lipid levels, smoking, hypertension, diabetes mellitus, abdominal obesity, psychosocial factors, low consumption of fruit and vegetables, alcohol abuse, and no regular physical activity account for most of the risk factors for myocardial infarction worldwide in both genders and all ages in all regions.^1^ The riskfactor distribution for coronary artery disease (CAD) in our sub-population may be similar to those in the Western world.^1^,^2^ Control of these risk factors is key to the prevention of and reduction in the incidence of CAD.^3^

Over the past 20 years, there has been considerable progress with improved outcomes in the treatment of acute coronary syndromes (ACS). These include the establishment of coronary care units and the development of antiplatelet therapy, refinement of anticoagulation strategies and introduction of fibrinolytic therapies. Percutaneous coronary intervention (PCI) has become the intervention of choice in the acute setting in ST-segment elevation myocardial infarction (STEMI). Early invasive intervention in non-ST-segment elevation myocardial infarction (NSTEMI) is also advocated.^4^-^8^

Randomised clinical trials in STEMI patients have shown that efficient triaging and early reperfusion therapy decreases mortality rates. Early thrombolysis is effective in improving outcomes in acute STEMI. Although timely performance of primary PCI is more effective in the restoration of patency, and for lower re-occlusion rates, improved residual left ventricular function and better clinical outcomes, this benefit diminishes with any delays.^5^,^9^

The initial strategy in NSTEMI is to alleviate ischaemia and symptoms by using anti-ischaemic agents, antiplatelets, anticoagulants, IIb/IIIa inhibitors, to monitor the patient with serial ECGs, and carry out repeat measurements of markers of myocardial necrosis. The invasive coronary approach has been shown to reduce mortality rates in NSTEMI patients.^10^ Research evidence has documented reduced mortality rates at 30 days with the use of β-blockers, ACE inhibitors, antiplatelet therapy and statins, smoking cessation, and timely reperfusion therapy in acute myocardial infarction.^11^,^12^

The GRACE risk model has been validated to establish the in-hospital mortality risk in patients with STEMI and NSTEMI. In this model, the risk factors predicting early mortality include age over 70 years, prior myocardial infarction, Killip class at admission, anterior myocardial infarction, and the combination of hypotension and tachycardia. The GRACE risk model assists in risk profiling and hence prioritising care.^13^,^14^

The Nairobi Hospital has a well-equipped emergency department and intensive care unit (ICU), and a modern cardiac catheterisation laboratory with well-trained staff. At the Nairobi Hospital, fibrinolytic therapy is the treatment of choice for STEMI. Primary PCI is increasingly used however it is not yet available timeously and consistently at all hours, due to various logistical factors.

This was a retrospective study of the Nairobi Hospital ICU and high-dependence unit (HDU) in-patient records to describe the demographics, risk factors, clinical characteristics, management and outcomes of patients diagnosed with acute myocardial infarction admitted to the Nairobi Hospital ICU and HDU from January 2007 to June 2009.

## Methods

The hospital ethics committee gave consent for the study.The in-patient patients’ records were retrieved and a cardiologist verified the diagnosis of STEMI and NSTEMI, after which the pre-specified data variables were retrieved and filled into a pre-designed study pro forma.

Patients who presented with a diagnosis of acute myocardial infarction and were over 18 years were included. STEMI was defined according to the European Society of Cardiology guidelines, as patients with acute chest pain and persistent (> 20 minutes) ST-segment elevation at the J-point in two contiguous leads with the cut-off points: ≥ 0.2 mV in men or ≥ 0.15 mV in women in leads V2–V3 and/or ≥ 0.1 mV in other leads. For the purposes of reperfusion strategy, true posterior myocardial infarction was considered in patients with ST-depression in the anterior leads or new prominent R waves on the same leads.^15^,^16^

NSTEMI was defined as patients with chest pain but without persistent ST elevation, new horizontal or down-sloping ST depression ≥ 0.05 mV in two contiguous leads; and/or T inversion ≥ 0.1 mV, flat T waves and pseudo-normalisation of T waves in two contiguous leads with prominent R wave or R/S ratio or no ECG changes at presentation. The confirmation of acute myocardial infarction was made based on elevated cardiac biomarkers.^15^,^16^ Significant atheromatous lesions were defined as a left main stem lesion of > 50% stenosis and lesions involving the left anterior descending, left circumflex and right coronary arteries > 70% stenosis.

We analysed the demographic data, mode of transport to the accident and emergency department, first medical contact, time of arrival after onset of chest pain, door-to-first ECG time, and ECG-to-cardiologist time. The GRACE risk score of each patient on admission was documented as per the parameters at admission.

The risk factors for coronary artery disease analysed included age > 55 years in men and > 65 years in women, male gender, current smoking, low high-density lipoprotein cholesterol (HDLC) level < 40 mg/dl (1.04 mmol/l), systemic hypertension (blood pressure > 140/90 or on antihypertensives), diabetes and family history of premature coronary artery disease.

The initial medication given at the accident and emergency department, the type of myocardial infarction and the reperfusion treatment for STEMI were noted. The door-to-needle time and fibrinolytic agent used were both recorded. The coronary anatomy and TIMI grade for coronary blood flow were documented as per the cardiologist’s notes and confirmed by an interventional cardiologist who studied the digital images of the coronary angiogram. The in-hospital complications, duration of hospitalisation, in-hospital deaths and discharge medications were noted.

## Statistical analysis

The extracted data were entered into the Statistical Package for Social ScienceTM (SPSS) version 13.0 for Windows (SPSS, Chicago, IL, USA) statistical software to check for errors and perform the requisite statistical tests. Data were analysed using the same software. Descriptive analysis was performed to characterise the number and type of patient outcomes. To obtain insight into the social demographic factors of the patients, frequency tables were used with accompanying percentages.

Bivariate comparisons of continuous symmetric characteristics, such as duration of time between onset of chest pain/ symptoms and arrival at hospital, door-to-first ECG time and ECG-to-cardiologist time were performed using the Student’s t-test and Mann–Whitney test for non-symmetric characteristics for patients with independent variables (in-hospital outcome). Fisher’s exact test and the chi-squared test, as appropriate,were used for comparison of categorical characteristics, such as gender, coronary artery anatomy and in-hospital complications with patients’ in-hospital outcomes.

Correlations between variables were tested using the Pearson’s correlation co-efficient. Prevalence of risk factors was calculated with accompanying 95% confidence intervals. Statistical significance was defined as a two tailed p-value ≤ 0.05.

## Results

Sixty-four patients fulfilled the criteria for STEMI and NSTEMI from January 2007 to June 2009 and 87.5% were male. The mean age was 56.7 years. Of the study population, 60.9% were from the community, while 26.6% were referrals from other health facilities; 28.1% were brought by ambulance but mode of transport was not documented in 67.2% of the cases. Five per cent of the patients were already hospitalised and 89.1% had their first medical contact in hospital. Among the patients, 17.2% arrived within one hour of onset of chest pain, whereas 40.6% arrived at the emergency department more than 12 hours after the onset of chest pain.

The presence of coronary artery risk factors in this population was as follows: systemic hypertension was found in 71.9% of patients, 42.2% were over 55 or 65 years in men and women, respectively, 35.9% were cigarette smokers, 25% had a low HDL-C level, 25% had diabetes mellitus, 8% had a documented family history of premature coronary artery disease, 18.8% hada previous history of acute coronary syndrome and 9.4% had chronic kidney disease.

The documented door-to-ECG time was less than 10 minutes in only 10.9% of patients and the ECG-to-cardiologist time was less than 30 minutes in 36.5%. Both aspirin and clopidogrel were received by 96.9% of patients on arrival at the emergency department. A loading dose of aspirin was given in 53.2% of patients, whereas 62.9% received a loading dose of clopidogrel; 89.1% received enoxaparin and only 9.4% received unfractionated heparin; 67.2% had transthoracic echocardiography during their hospital stay, of whom 76.9% had a left ventricular ejection fraction (LVEF) > 40%.

For type of acute myocardial infarction, 60.9% had a diagnosis consistent with STEMI and 39.1% had NSTEMI. In the NSTEMI arm, 44% of the patients received IIb/IIIa inhibitors, mainly eptifibatide infusion, and 68% had a coronary angiogram done before hospital discharge. The coronary anatomy was consistent with significant atheromatous lesions in 79% of the patients. Of those who had no coronary angiography, 60% had non-invasive testing for myocardial ischaemia before discharge.

In the STEMI arm, 79.5% of patients received thrombolysis, 17.9% underwent rescue PCI and 2.6% did not receive reperfusion therapy. None of the patients underwent primary PCI. The door-to-needle time was less than 120 minutes in 45.2% of the thrombolysis patients and 38.7% had no documentation of door-to-needle time. The thrombolytic agent was tenecteplace in 80.6% and steptokinase in 6.5% of patients. Only 51.6% of the thrombolysis patients had an ECG done at 90 minutes post thrombolysis, of whom 56.3% had achieved reperfusion. Of the patients who did not achieve reperfusion, 66.7% underwent rescue PCI. The coronary anatomy was consistent with significant atheromatous lesions in 82.1% of the patients who underwent angiographic studies.

Of the patients who underwent coronary angiography, 29% were managed medically, 19.1% were referred for coronary artery bypass grafting (CABG), and 40.4% had PCI with 84.2% receiving drug-eluting stents. Of the patients who underwent PCI, 87.5% achieved TIMI flow of grade 2 to 3.

Cardiogenic shock occurred in 17.2% of patients, new atrial fibrillation in 6.3% and cardiac arrest in 3.1%. Sustained ventricular tachycardia or ventricular fibrillation occurred in 5.3%, atrioventricular block in 4.7% and acute kidney injury (creatinine > 200 μmol/l) in 6.3%.

The mean duration of hospitalisation was 6.69 days. In-hospital mortality rate was 9.4%. The mean in-hospital probability of death according to the GRACE risk score was 16.05%.

Upon discharge from hospital, 84.5% were prescribed β-blockers, 48.3% were on ACE inhibitors or angiotensin receptor blockers (ARBs), 96.6% were on low-dose aspirin, 96.6% on clopidogrel and 93.1% on statins.

## Discussion

Cardiovascular risk factors have been determined from several landmark studies, mostly performed in the Western world. The African forum has recently embarked on some local studies.

The patient population in our study was similar in age and gender to that of other African studies on traditional cardiovascular risk factors in patients with confirmed coronary artery disease.^2^,^17^ The most prevalent risk factor in our study was systemic hypertension, which reflects the findings in previous studies. In the overall INTERHEART study, 39% of the patient population had systemic hypertension, whereas in the INTERHEART Africa arm, it was reported in 42.3% of cases.^1^,^2^,^17^,^18^

Emergency medical systems were not in place in our local setting at the time we carried out this study, therefore first medical contact was at the emergency department. Most patients arrived after three hours from onset of chest pain. The initial triage at the emergency department was not optimal with regard to door-to-ECG, ECG-to-cardiologist and door-to-thrombolysis times going beyond the recommended door-to-needle time of 30 minutes. The hospital is currently looking at a system that will allow for immediate interactive communication with the concerned cardiologist to advise on the management of these patients.

The thrombolytic agent used most frequently was tenecteplace. Almost all patients received antiplatelet and antithrombin co-therapy as per the current guidelines. The gold standard of acute management of ST-elevation myocardial infarction is primary PCI, with thrombolytic therapy being effective when given early. Thrombolysis was the standard approach in this study.

Most patients in our study had an echocardiogram performed during their hospital course. It is generally indicated that echo assessment of cardiac anatomy and function be done in the first 24 to 48 hours after acute myocardial infarction.^4^

Our data showed that the in-hospital mortality rate of patients with a diagnosis of acute myocardial infarction was 9.4%. Despite the small size of the study population, this is comparable to data derived from European studies. The mean probability of in-hospital death in our study according to the GRACE risk score was 16.05%.

Secondary preventive medication was prescribed for most patients as per the standard recommendations. However ACE inhibitors or ARBs were prescribed in less than 50% of patients.

## Conclusion

The risk-factor assessment in our population of patients, albeit small, was in keeping with traditional risk factors for coronary artery disease found in other studies. The risk for acute myocardial infarction was found to increase with higher income and educational level in our black African population, in contrast with findings in other African groups. With advances in the field of cardiology, the local emergency medical system will improve and timely invasive management of patients presenting with acute myocardial infarction will be available. Currently, there is room for improvement in reconciling the gap between actual and recommended patient care. There is also a need to develop local management protocols for patients with acute myocardial infarction, based on local specialist experience and the available facilities. The Nairobi Hospital is committed to putting in place facilities to allow for primary PCI and early thrombolysis.
